# Evaluation of symmetry behavior of surgically assisted rapid maxillary expansion with simulation-driven targeted bone weakening

**DOI:** 10.1007/s00784-021-03958-w

**Published:** 2021-05-05

**Authors:** S. Chhatwani, K. Schudlich, S. C. Möhlhenrich, A. Pugachev, A. Bicsak, B. Ludwig, S. Hassfeld, G. Danesh, L. Bonitz

**Affiliations:** 1 Department of Orthodontics, Faculty of Health, University of Witten/Herdecke, Alfred-Herrhausen-Str. 45, 58455 Witten, Germany; 2 CADFEM Medical GmbH, Marktplatz 2, 85567 Grafing, Germany; 3 Department of Cranial and Maxillofacial Surgery, Faculty of Health, University of Witten/Herdecke, Klinikum Nord, Münsterstr. 240, 44145 Dortmund, Germany; 4 Private orthodontic clinic, Am Bahnhof 54, 56841 Traben-Trarbach, Germany

**Keywords:** Surgically assisted rapid maxillary expansion, CBCT, Asymmetry, 3D superimposition, Transpalatal distractor

## Abstract

**Objectives:**

Surgically assisted rapid maxillary expansion (SARME) is a treatment modality to overcome maxillary constrictions. During the procedure of transverse expansion, unwanted asymmetries can occur. This retrospective study investigates the transverse expansion behavior of the maxilla utilizing a simulation-driven SARME with targeted bone weakening.

**Materials and methods:**

Cone beam computer tomographies of 21 patients before (T1) and 4 months after treatment (T2) with simulation-driven SARME combined with a transpalatal distractor (TPD) and targeted bone weakening were superimposed. The movements of the left, right, and frontal segments were evaluated at the modified WALA ridge, mid root level, and at the root tip of all upper teeth. Linear and angular measurements were performed to detect dentoalveolar changes.

**Results:**

Dentoalveolar changes were unavoidable, and buccal tipping of the premolars (6.1° ± 5.0°) was significant (*p* < 0.05). Transverse expansion in premolar region was higher (6.13 ± 4.63mm) than that in the molar region (4.20 ± 4.64mm). Expansion of left and right segments did not differ significantly (*p* > 0.05).

**Conclusion:**

Simulation-driven SARME with targeted bone weakening is effective to achieve symmetrical expansion in the transverse plane.

**Clinical relevance:**

Simulation-driven targeted bone weakening is a novel method for SARME to achieve symmetric expansion. Dental side effects cannot be prohibited.

## Introduction

Maxillary constriction is a malocclusion with a prevalence of 8 to 10%, which can be observed in adolescents and adults [1, 2]. Clinically, it is not only manifested in narrowed nasal cavities, arch length discrepancies, and anterior crowding but is usually also associated with a posterior crossbite occurring unilaterally or bilaterally [3, 4].

Studies have shown that the posterior crossbite is one of the most common dental malocclusions with a prevalence of 8 to 22% [5].

Once diagnosed, crossbites, whether dental or skeletal, should be treated, as they do not only lead to aesthetic problems but also influence function of the temporomandibular joint in the sense of incorrect loading and can lead to negative effects on body posture [6].

The main goal of treatment of skeletal crossbites caused by maxillary constrictions is to achieve a transverse skeletal expansion of the maxilla with the least possible dental influence, in order to achieve harmony of the lower and upper dental arches and to eliminate the discrepancies.

In order to achieve this treatment goal, Haas presented a tooth- and tissue-borne appliance consisting of molar bands that physically grasp the teeth assisted by two plastic bases that are connected medially by a screw [7].

This device was optimized by Biedermann in 1968, with an all wire frame and a jackscrew, known as the tooth-borne Hyrax expander [8].

Dentally anchored appliances are often accompanied by undesirable periodontal and orthodontic side effects, such as buccal tilting of teeth, root resorptions, cortical fenestration, and orthodontic relapses [9, 10]. Mommaerts described the transpalatal distractor (TPD) in 1999, which consists of two telescopic cylinders and is anchored solely to the bone, thus ensuring skeletal force transmission. Anchoring and force transmission directly to the bone also allows not only for greater but also extremely efficient palatal expansion [10, 11]. For the sake of completeness, it should be mentioned that Hybrid Hyrax appliances, which are both skeletally and dentally anchored, can also be used for palatal expansion [12].

The midpalatal suture is subject to a relatively long process of ossification and is not yet ossified in childhood. In adults, on the other hand, it has numerous interlocked bone bridges, which are so heavy that separation of the two halves of the upper jaw is only possible with fracturing this interdigitation [13].

Opinions differ regarding age and the need of surgical assistance for this procedure. Epker and Wolford recommend a surgically assisted rapid maxillary expansion (SARME) after reaching the age of 16 [14]. Timms and Vero, on the other hand, claim that the maximum age for a conventional SARME should be 25 years [15].

Alpern and Yurosko also differed in their study by gender and concluded that SARME is indicated for men at over 25 years of age and for women at over 20 years of age [16].

A classification for midpalatal suture maturation utilizing cone beam computer tomography (CBCT) has been described by Angelieri et al. and shows that complete fusion of the midpalatal suture can already occur in females aged between 14 and 17 years [17]. In cases of completed mineralization with closure of the midpalatal suture, the use of SARME is advocated [18].

An increasing weakening of the bony pillars seems to affect the expansion behavior of the maxilla [19]. Especially, the zygomaticomaxillary junction plays a major role in resistance to expansion, and therefore, a corticotomy is performed from the piriform aperture to the pterygomaxillary junction. Some surgeons release the midpalatal suture to improve mobility, and also, a pterygoid disjunction seems to affect the pattern of maxillary expansion [20, 21]. But its release is not preferred by all surgeons, [18] and its effect in context of orthodontic treatment is less [22].

Postoperative examination of the maxilla or biomechanical anatomic models show that expansion of the maxilla after SARME is often asymmetrical [23, 24]. Elkenawy et al. show asymmetric expansions in more than 50% of their sample group [25]. These asymmetries can also occur in oblique direction [26]. It has been reported that in cases of asymmetric expansion, an additional corrective surgery can be necessary [27]. A possible explanation besides other factors leading to these asymmetries could be differential bone density at the sutures and their surrounding structures [25].

In order to counteract these asymmetries, a simulation-driven surgical therapy with targeted bone weakening was performed for SARME. This study with its retrospective character investigates the transverse expansion behavior of the maxilla with this technique.

## Material and methods

Data of all patients of the Department of Cranial and Maxillofacial Surgery, in Dortmund, who underwent a SARME in the period from May 2006 to June 2017 as part of an interdisciplinary orthodontic-surgical treatment, were examined for symmetry regarding the expansion procedure.

Inclusion criteria for this retrospective cohort study without control group were the availability of CTs or CBCTs before and after SARME and the presence of a maxillary transverse deficiency which was to be solved by surgically assisted rapid maxillary expansion utilizing a bone-borne transpalatal distractor (RPE, Gebrüder Martin GmbH & Co. KG, Tuttlingen, Germany). All cases had to be operated by the same workflow with simulation-driven 3D planning and guide construction.

Patients with tooth extraction, tooth osteotomy, tumors, syndromal diseases, traumatic injuries, and severe craniofacial anomalies were excluded from the study. Patients with tooth-borne appliances or with CBCTs showing artifacts which prohibited the analysis were not eligible for this study. A total of 75 patients from 96 patients screened did not fulfill the inclusion criteria.

Total sample size consisted of 21 patients. The study group consisted of female and male patients. Data of patients were anonymized after collection.

Ethics approval for this study was given by the ethics committee of the University of Witten/Herdecke, Germany (application number 03/2019).

### Finite element simulation

With the help of individual CT- or CBCT-based DICOM data, both the spatial configuration and the thickness of the remaining bone were analyzed and transferred into the simulation software to perform the preoperative “virtual osteotomy.”

**Fig. 1:**
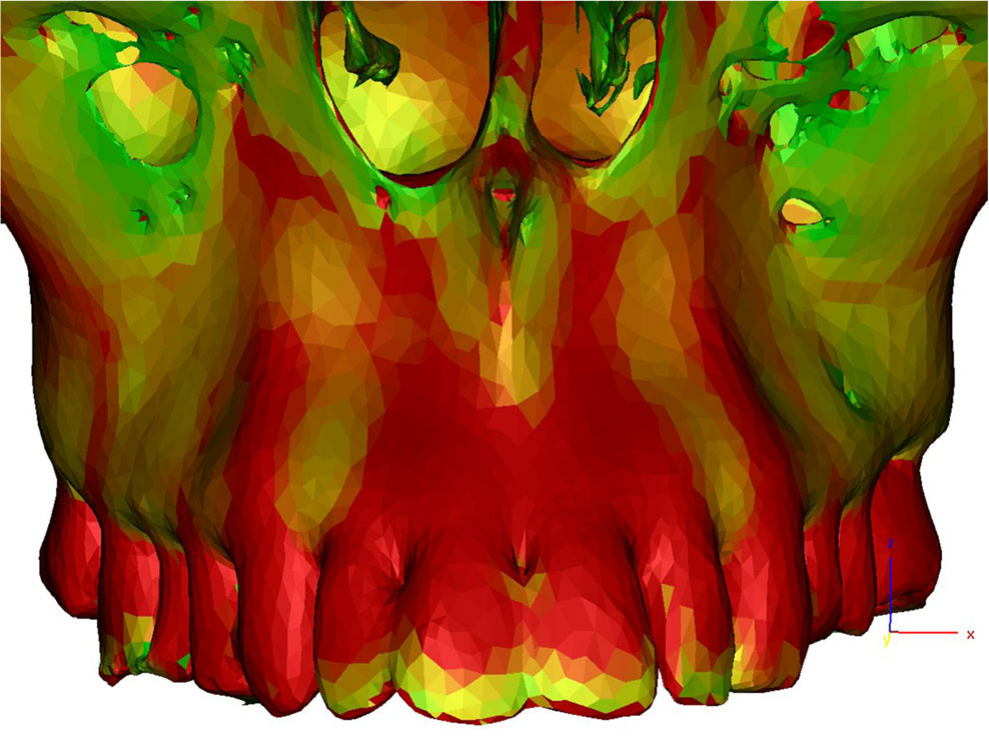
Segmented maxilla with color-coded bone thickness (green, thin bone layer; red, thick bone layer)

Three-dimensional finite element analysis (FEA) was used to examine deformation and stress-strain response of the anatomical model of the skull under the applied osteotomies and the distractor load before the surgery. The skull was segmented with MIS software (Mimics Innovation suite V20.0, Materialise NV, Leuven, Belgium) which allows analysis of individual differences of bone thickness (Fig. 1).

For finite element analysis, ANSYS Mechanical APDL 19.2 (ANSYS, Inc., Canonsburg, PA, USA) was used. The STL model of the individual skull with a specific osteotomy configuration was imported into ICEM CFD (ANSYS, Inc., Canonsburg, PA, USA) for meshing. Tetrahedral unstructured grids were generated in ANSYS ICEM CFD 19.2 based on the STL geometry of the skull.

**Fig. 2:**
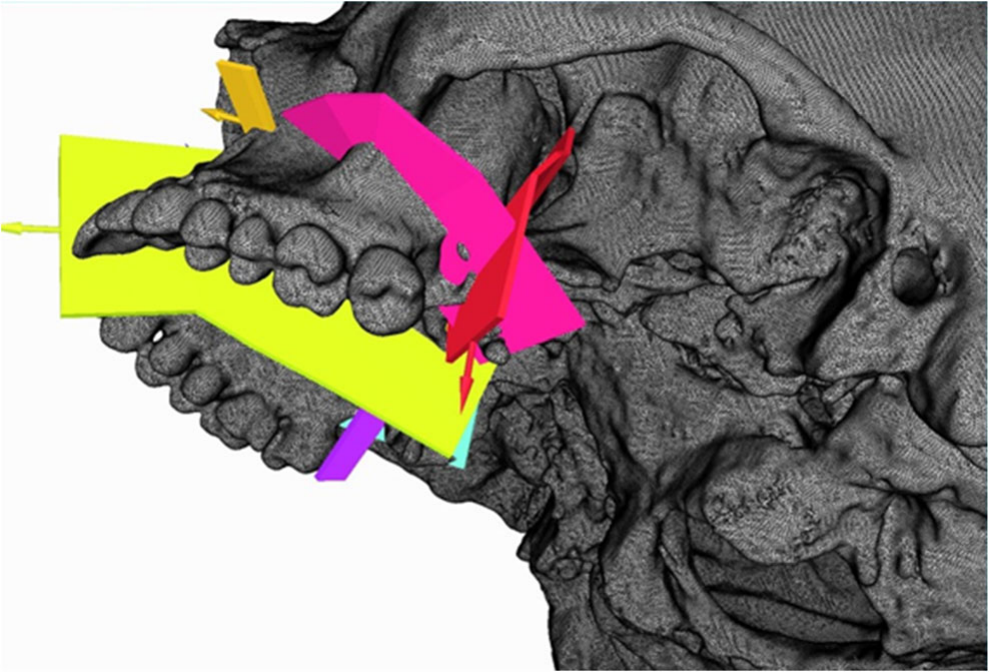
Insertion of individual planes for osteotomy

A partially or complete opening of the lateral sinus wall and disconnection of the midpalatal suture, as advised by Lines, could be performed individually in the software environment (Fig. 2). Mesh refinement and mesh smoothing were generated to ensure a high finite element quality. An input file with the FE mesh was exported for the solver. The tetrahedral mesh file was imported into the ANSYS Mechanical APDL software application. With the programming language APDL (ANSYS Parametric Design Language), the definition of material properties and boundary conditions was performed. For the bone material, Poison’s ratio of 0.3 and a modulus of elasticity of 15000MPa are defined. The transversal distraction of both palatal halves was simulated by applying a force of 150N at the endpoints of the distractor which were individually adjusted.

**Fig. 3:**
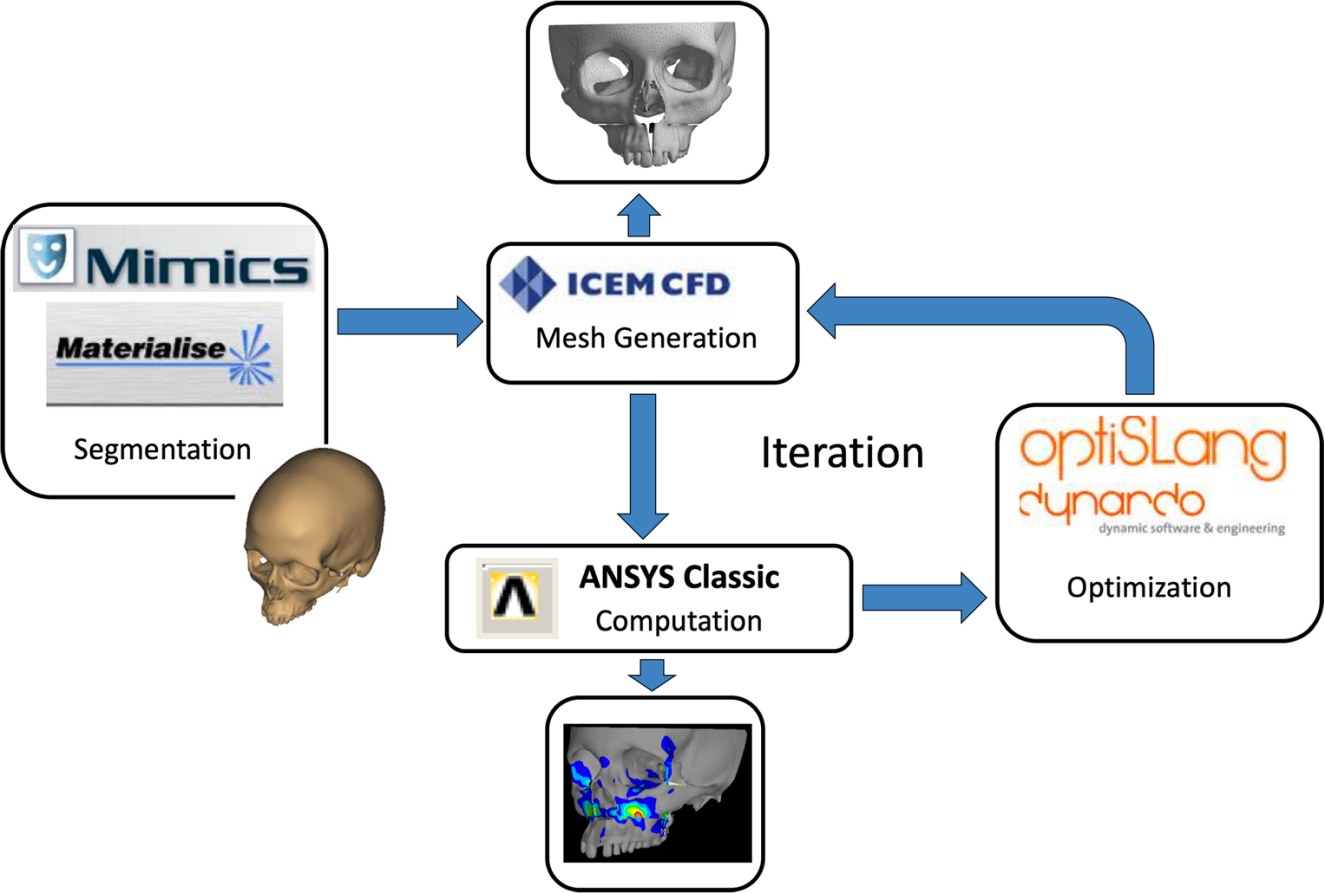
Complex workflow for bone segmentation and statistical analysis

The whole workflow with the meshing of bone geometry, the finite element model generation, and the simulation was performed automatically (Fig. 3).

The strain and stress plots and the deformation were exported for visual inspection. A sampling and statistical analysis was performed using optiSLang 7.2.0 (Dynardo GmbH, Weimar, Germany). The design variables were defined by the surgeon. To generate a large number of designs for the evaluation, the Latin hypercube sampling method (LHS) was applied. It describes a statistical method for generating a near-random sample of parameter values from a multidimensional distribution. Between the ANSYS software and the optiSLang software, an interface for transfer of values was implemented.

A symmetrical expansion of the maxilla is favored by a small difference in the stiffness of the maxillary bone of the left and right sides. This value is assumed to be the asymmetry criterion based on biomechanical principles. The smaller the difference, the more symmetrical expansion can be achieved.

For reliability and optimization of computational results, a minimum number of 100 iterations was performed. The resulting osteotomy configuration was critically evaluated by the surgeon.

**Fig. 4:**
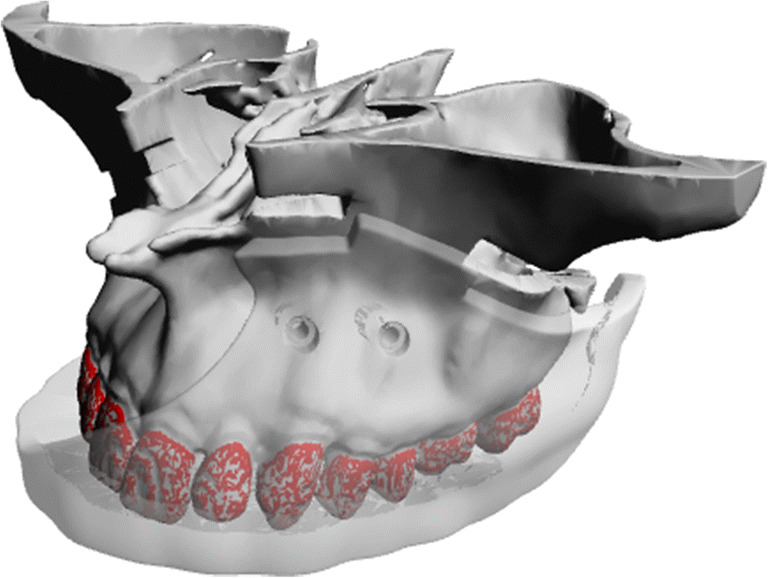
Design of a surgical guide to transfer the osteotomy on the bone surface

The simulation results were then used to create an individual surgical guide (ANSYS SpaceClaim software ANSYS Inc., Canonsburg, USA) for the left and right sides via CAD/CAM technique and subsequently milled (Hermle C30U, Maschinenfabrik Berthold Hermle AG, Gosheim, Germany) from PEEK (polyetheretherketone) [28, 29] (Fig. 4).

### Surgical procedure

All surgical procedures were carried out by the same surgeon.

**Fig. 5:**
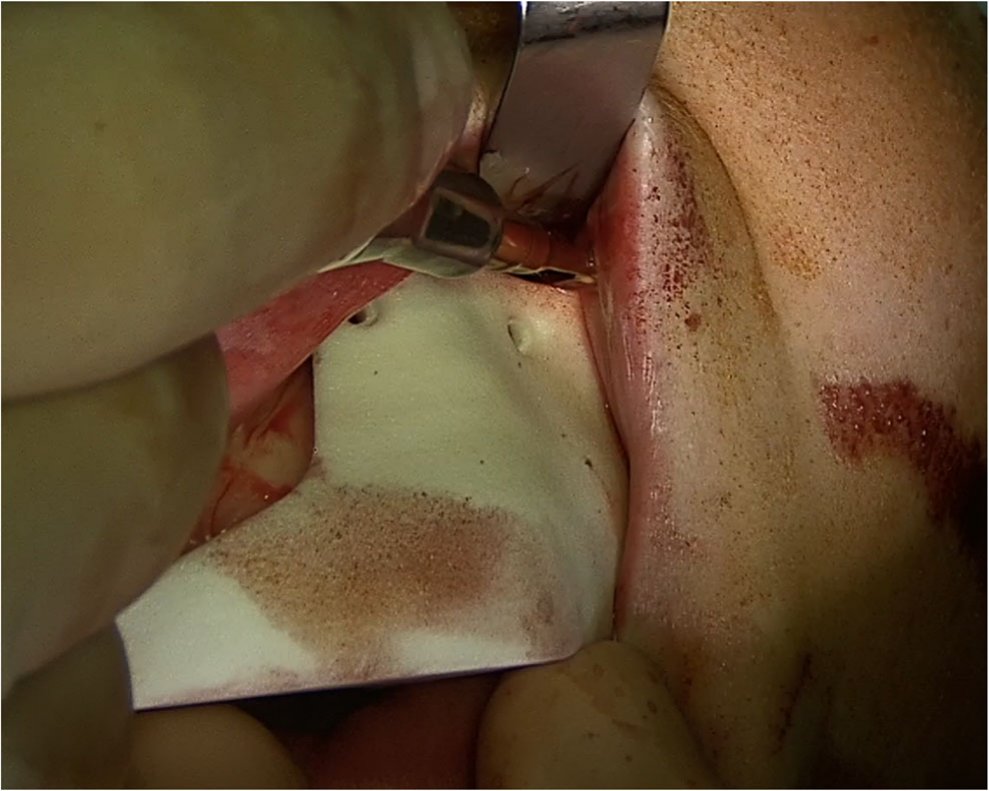
Introperative insertion of the surgical guide

The osteotomy was performed piezosurgically at the Le Fort I level. Based on Line’s technique of osteotomy from 1975, this modified procedure leaves iatrogenic bone bridges in the area of the canines due to their pronounced root length [30]. After anesthesia, the incision of the mucosa was made paramarginally, vestibular from region 13 to 16 and region 23 to 26, with tunnelation anteriorly and posteriorly. The submucosal and subperiosteal preparation in tunnel technique extends into the infraorbital region as well as via the zygomaticoalveolar crest to the retrotubar maxillary region and into the pterygopalatine fossa. The surgical guide for one side was then inserted (Fig. 5). Subesquent osteotomy of the vestibular wall of the maxillary sinus in accordance with the recesses of the respective surgical guide (right vs. left) was performed. The bone incisions through the maxillary bone were started laterally following the guide with a piezosurgery approach at the zygomatic insertion, and after opening the maxillary sinus, they were moved medially through the facial maxillary sinus wall and the nasal aperture. The pterygomaxillary separation was performed with a special extremely curved Kawamoto chisel from the dorsal tunnel edge, followed by vertical incision of the upper lip frenulum and opening of the midpalatal suture piezosurgically and with the chisel. The distractor was inserted at the pre-planned position via a guide in the palate with a horizontal incision and anchored with a fixation screw on each side. Wound closure was undertaken with suturing. The medial and dorsal walls of the maxillary sinus remain intact during the osteotomy. The TPD was checked for proper function by opening the device to 3mm. Then, the TPD was turned back to 1mm of opening. After a short healing time, the appliance was activated daily by the patient by 0.5–1mm until desired expansion was achieved.

### 3D Superimposition and measurements

All CBCT scans were made with Sirona GALILEOS and GALILEOS plus (Dentsply Sirona, Bensheim, Germany).

The CBCTs were collected before (T1) and 4 months after (T2) the surgical procedure with a slice thickness of 0.3mm. The output data, a DICOM file, was converted into a reconstructed, virtual, three-dimensional model (STL file) with the software InVesalius 3.1 (CTI, Brazil), and an area of interest was selected, and the virtual object was then cropped in the same software. The STL data files for T1 and T2 for each patient were then transferred to the 3D inspection software GOM Inspect V7.5 (GOM GmbH, Braunschweig, Germany), which was used to superimpose the three-dimensional models

After automatic pre-alignment, a local best fit was performed to superimpose T1 and T2 data by a defined bony area of the anterior cranial base. Anatomical landmarks were the greater wings of the sphenoid bone and the sella turcica.

In order to examine the symmetry obtained in the transversal plane during expansion by means of a targeted bone weakening, 3D surface distance comparisons were carried out at predefined points at level of the teeth from 16 to 26.

**Fig. 6:**
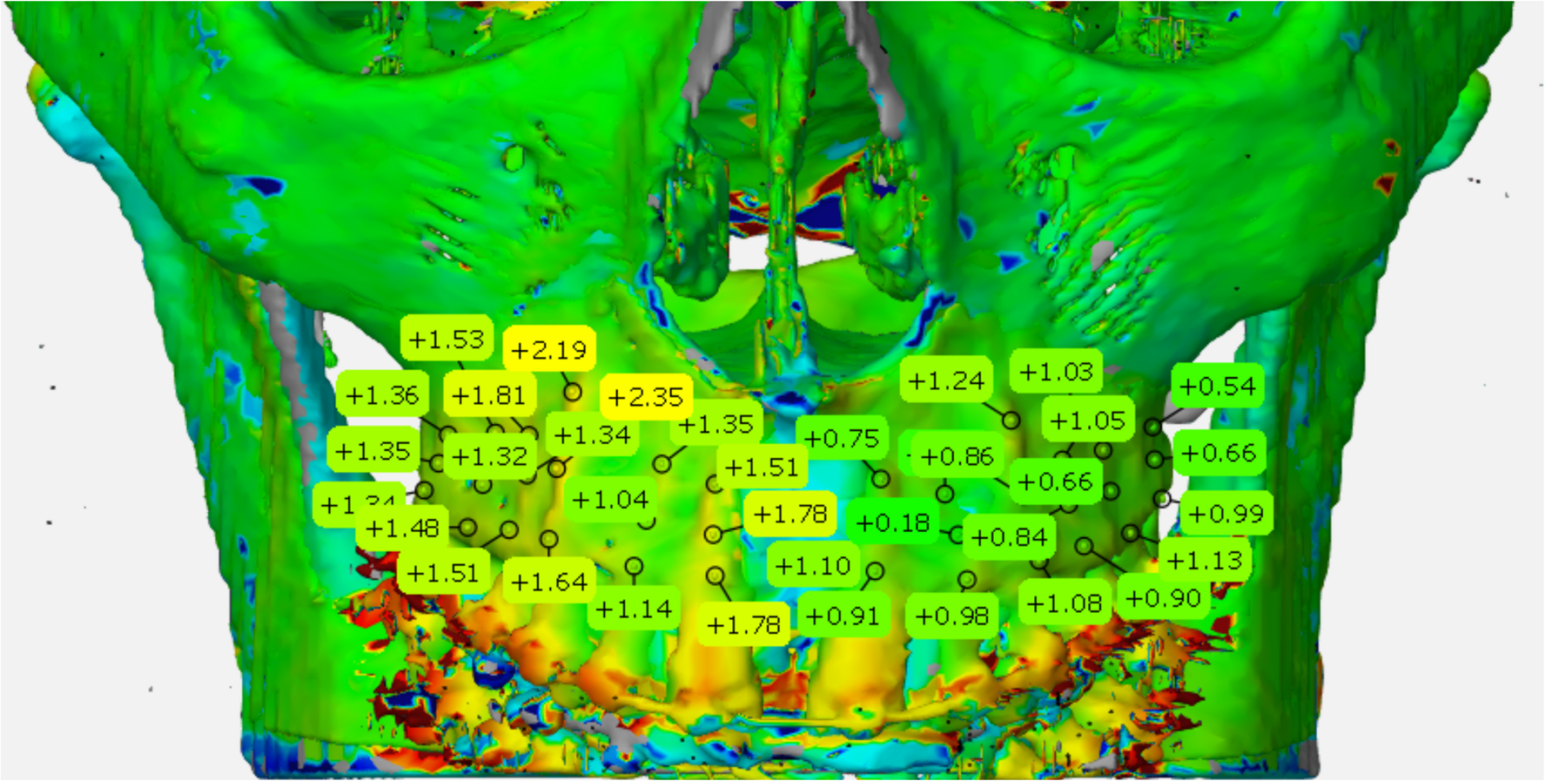
Surface distance comparison of superimposed CBCTs from T1 and T2 with defined maxillary landmarks

These points include a modified WALA Ridge, the root tip, and a point in the middle third of the root on a line connecting the modified WALA Ridge and root tip (Fig. 6).

The WALA Ridge is an anatomical feature coronal to the mucogingival junction but refers to soft tissue and to the mandible [31]. The modified WALA Ridge is defined in this study as the thickest hard tissue landmark in an area coronal of the assumed mucogingival junction of the maxilla.

In general, 36 points should be measured per patient if no teeth were missing.

Lengths and angles were also measured with RadiAnt DICOM Viewer (Medixant, Poznan, Poland).

**Fig. 7:**
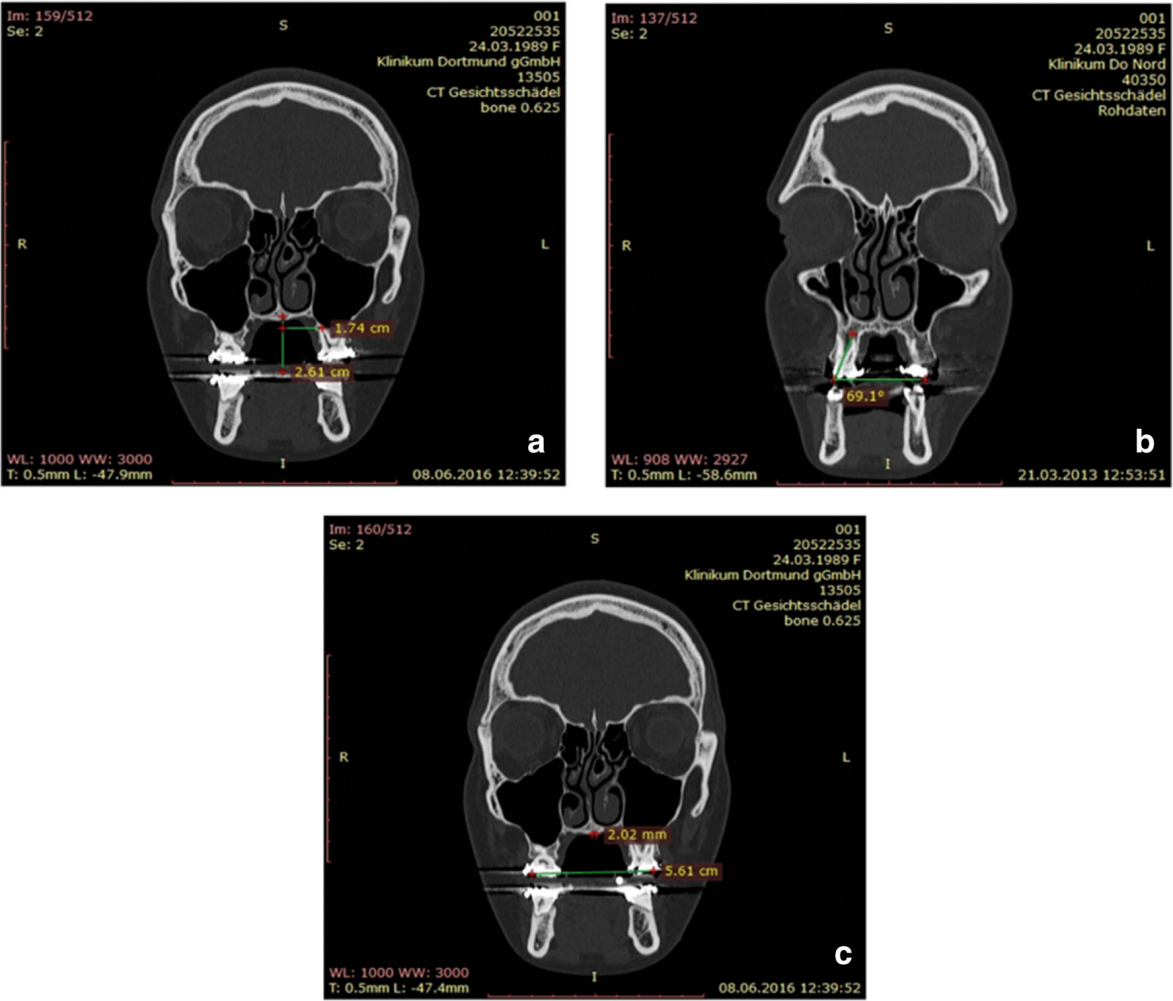
**a** Measurement from a reference line at the midpalatal suture to the palatal root tip of the upper molar. **b** Angular measurement of the upper molar to the occlusal plane. **c** Transverse width measurement from buccal cusp to buccal cusp of the upper molars

At first, the midpalatal suture was identified, and a vertical line was drawn through it in the coronal plane so that this line could be used as reference. Subsequently, the distance of the palatal root tips of the upper molars (16 and 26) and the first bicuspids (14 and 24) was measured to this reference line (Fig. 7a.). In addition, the angle between the palatal root tip of the same teeth and the occlusal plane was measured (Fig. 7b). Finally, the transverse width between the buccal cusp tips of 16 and 26 and 14 and 24 was measured (Fig. 7c).

All acquired data were entered in a spreadsheet (Excel 2019, Microsoft Corporation, Redmond, USA) and transferred into a statistics software MEDAS IT (C Grund, EDV Systems, Margetshoechheim, Germany). Descriptive statistics and Wilcoxon pairwise comparison for dependent samples were performed. Additionally, a cluster analysis according to Ward [32] and pairwise comparison for independent samples in the form of Mann-Whitney *U*-tests were performed to analyze differences by type of cluster. For evaluation of statistical differences in left/right asymmetry of the clusters, two-factorial analysis of variance with repeated measurements on one factor was used.

Even though all patients were evaluated in general, some had to be excluded from certain measurements due to insufficient image quality of the CBCTs or due to absence of teeth.

## Results

### Transverse width changes at buccal cusps

The distance of the width of the buccal cusp tips between 14 and 24 differs significantly from T1 to T2 (*p*<0.001). The mean difference was 6.13mm ± 4.6mm.

Also, the transverse width in the area of the first molars was significantly different when comparing T1 and T2 (*p*< 0.01) with a mean difference of 4.2mm ± 4.64mm (Table 1).

**Table 1:** Wilcoxon test for two dependent samples comparing transverse width at T0 and T1 (*n* sample size, *SD* standard deviation)

Transverse width							
Teeth	Time	*n*	Mean (mm)	SD	Mean of difference (mm)	SD of difference (mm)	Wilcoxon *p*
14–24	T1	20	39.6	4.9	6.13	4.63	
14–24	T2	20	45.7	2.7			0.00024***
16–26	T1	19	50.9	5.3	4.2	4.64	
16–26	T2	19	551	3.1			0.004**

* = *p* < 0.05; ** = *p* < 0.01; *** = *p* < 0.001

### Distance of root apex to midpalatal suture

The distances of the apices of the upper first premolars and upper molars to the midpalatal suture show significant changes from T1 to T2 (*p*<0.05).

A mean increase in distance of the apex of 14 to the midpalate suture by 0.97mm ± 1.69mm was to be seen whereby the distance of the root of 24 showed an increase of 1.96mm ± 1.55mm.

The distances of the apices of the first upper molars showed an increase of 1.38mm ± 1.41mm on the right side and by 1.26mm ± 1.48mm on the left side (Table 2).

**Table 2:** Transverse width changes at apices of teeth from T1 to T2 and Wilcoxon dependent pairwise comparison (*n* sample size, *SD* standard deviation)

Distance Tooth		*n*	Mean (mm)	SD	Mean of difference (mm)	SD of difference (mm)	Wilcoxon *p*
14	T1	20	16.5	2.9	0.97	1.69	
	T2	20	17.4	2.4			0.026*
16	T1	19	18.4	3.5	1.38	1.41	
	T2	19	19.8	3.2			0.00072***
24	T1	20	16.2	2.5	1.96	1.55	
	T2	20	18.2	2.3			0.0001***
26	T1	20	19.0	3.9	1.26	1.48	
	T2	20	20.3	3.7			0.0034**

* = *p* < 0.05; ** = *p* < 0.01; *** = *p* < 0.001

Angular changes of teeth to the occlusal plane were measured at time T1 compared to time T2 for the upper first premolars and first molars. These results show significant differences changes in angular changes change at time from T1 to T2 at for all teeth (*p*<0.05). Generally, the angles measured became more acute with the highest changes for tooth 14 (–6.11° ± 5.02°) and lowest change in angular measurement for 16 (–2.93° ± 4.58°) (Table 3).

**Table 3:** Angular changes of the tooth axis to the occlusal plane in degrees (*n* sample size, *SD* standard deviation)

Tooth	Time	*n*	Mean (degrees)°	SD	Mean of difference (degrees)°	SD of difference (degrees)°	Wilcoxon *p*
14	T1	20	80.23	8.44	–6.11	5.015	
	T2	20	74.12	7.05			0.0002***
16	T1	19	71.65	10.35	–2.932	4.583	
	T2	19	68.72	9.63			0.019*
24	T1	20	79.43	8.24	–3.145	6.788	
	T2	20	76.29	5.27			0.038*
26	T1	20	73.96	11.01	–3.69	5.973	
	T2	20	70.27	11.69			0.013*

* = *p* < 0.05; ** = *p* < 0.01; *** = *p* < 0.001

### Movement of segments

The maxilla was divided into three segments, namely a right (13, 14, 15, 16), frontal (12, 11, 21, 22), and left (23, 24, 25, 26) segment. The median value of all surface measurement points for each of these three segments was determined.

Subsequently, mean values of the median values of each expanded segment were determined, resulting in a mean value of 0.18mm ± 1.37mm for the frontal segment, 1.67mm ± 1.28mm for the left segment, and 1.14mm ± 1.09mm for the right segment.

The Wilcoxon test indicated statistically significant differences in amount of movement of the frontal segment compared to the left (*p*<0.001) and the frontal segments compared to the right segment (*p*<0.01). The left and right segments moved more than the frontal segment. Analyzing the cases globally shows a statistically insignificant difference in amount of movement between the left and right segments (*p*>0.05) (Table 4). The movement of the frontal segment was evaluated for the sake of completeness.

**Table 4:** Comparison of the segments utilizing the Wilcoxon test (*n* sample size, SD standard deviation)

Segment	*n*	Mean (mm)	SD	Mean of difference (mm)	SD of difference (mm)	Wilcoxon *p*
Frontal	21	0.18	1.37	–1.49	1.28	0.00015***
Left	21	1.67	1.28			
Frontal	21	0.18	1.37	–0.97	1.28	0.0035**
Right	21	1.14	1.09			
Left	21	1.67	1.28	0.53	1.17	**0.068**
Right	21	1.14	1.09			

* = *p* < 0.05; ** = *p* < 0.01; *** = *p* < 0.001

### Cluster analysis

A cluster analysis of all 36 position deviations was carried out to group the patients regarding similarities. Cluster 1 incorporates 15 patients that showed more movement of the segments than the other 6 patients which were grouped into cluster 2.

To assess significant differences, the median values of the front, left, and right segments between the two clusters’ *U*-test according to Mann and Whitney was applied.

The median value for movement of the frontal segment of cluster 1 was 1.03mm (68% CI 1.64 to –0.56mm) and differed significantly (*p*<0.05) compared to –0.71mm (68% CI 0.25 to –2.31mm) for cluster 2.

For the movement of the left segment of cluster 1, a median value of 1.71mm (68% CI 3.38–1.18mm) was noted and for cluster 2 a value of 0.61mm (68%CI 1.02–0.16mm). The difference between the left segments was significant (*p*<0.01). The comparison of the right segment of cluster 1 with a median value of 1.68mm (68% CI 2.43–1.04mm) and –0.23mm (68% CI 0.45 to –0.72mm) showed significant differences (*p* ≤ 0.001) (Table 5).

**Table 5:** Median comparison between cluster 1 and cluster 2 with Mann-Whitney *U*- test (*n* sample size, *SD* standard deviation, *CI* confidence interval)

Segment		*n*	Mean (mm)	SD	Median (mm)	68% CI		*U*-test *p*
Frontal	Cluster 1	15	0.668	1.02	1.030	1.637	–0.558	
	Cluster 2	6	–1.044	1.438	–0.705	0.254	–2.311	0.011*
Left	Cluster 1	15	2.114	1.208	1.710	3.375	1.184	
	Cluster 2	6	0.561	0.599	0.605	1.023	0.155	0.0028**
Right	Cluster 1	15	1.689	0.658	1.680	2.431	1.044	
	Cluster 2	6	–0.218	0.67	–0.230	0.451	–0.722	0.00015***

* = *p* < 0.05; ** = *p* < 0.01; *** = *p* < 0.001

It is noticeable that although no significant differences were measured in the median comparison of the left and right segments (Table 4), a median comparison of the right segment shows significant differences between the two clusters (Table 5).

Furthermore, a two-factorial analysis of variance with repeated measurements on one factor was performed to compare expansion symmetry between left and right segments.

While the right and left segments did not differ significantly from each other in regard to their movement (*p* = 0.53), a very significant difference was observed between clusters 1 and 2 (p = 0.0013), showing that cluster 1 had a higher amount of movement in both segments than cluster 2. An additional comparison within each cluster regarding left and right symmetry showed no significant changes (*p* = 0.073).

**Fig. 8:**
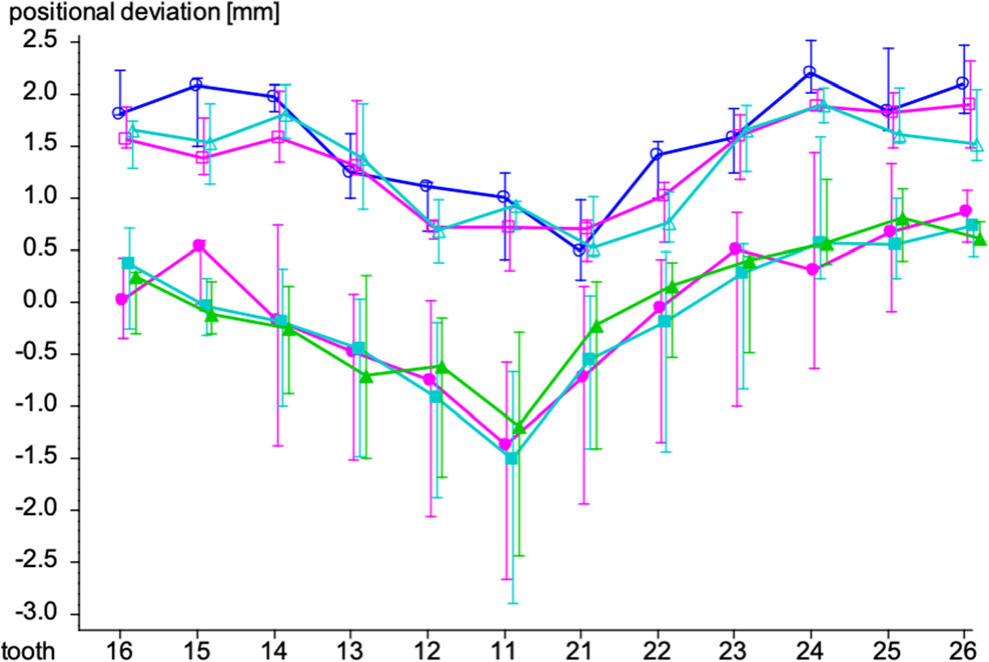
Graphical representation of the median values with 68% CI of cluster 1 (C1) and cluster 2 (C2) for the positional deviation at level of modified WALA, middle third of root, and root tip for each tooth. , mod WALA C1; , mid root C1; , root apex C1; , mod WALA C2; , mid root C2; , root apex C2

Fig. 8 shows the median values of the skeletal landmarks assessed at each tooth respecting the cluster grouping. It is visible that cluster 1 experienced more expansion than cluster 2 and that the curves are nearly symmetrical for the left and right side (Fig. 8).

**Fig. 9:**
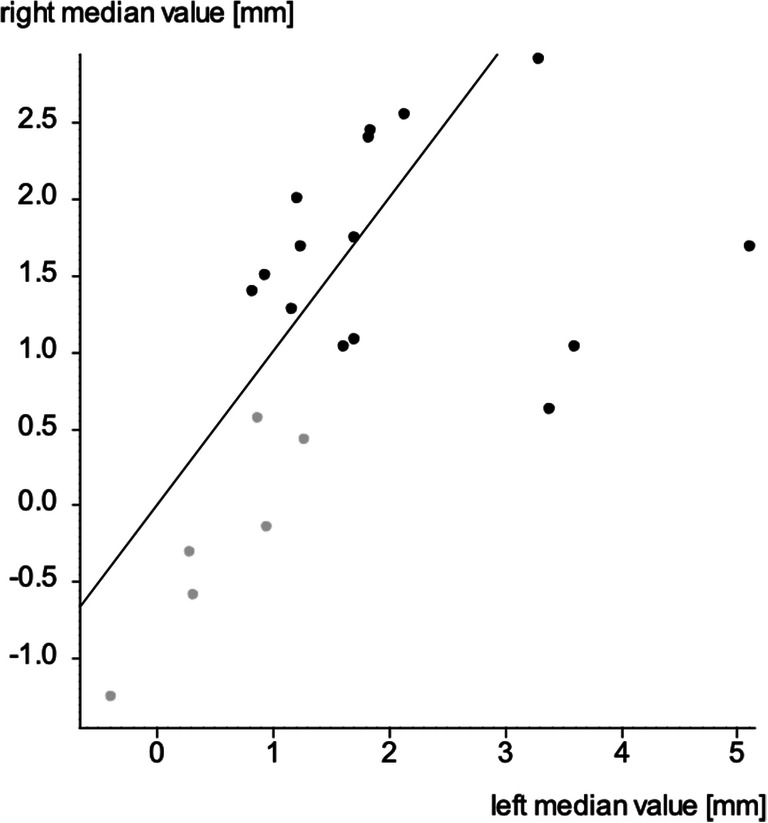
Graphical display of symmetry of expansion, each dot representing a patient with the right and left median value. The line represents the line of equal values. ●, cluster 1; , cluster 2

To evaluate for asymmetric expansion for each individual case, the median value of the left and the right segments for each patient can be used in a graphical representation. The straight line represents the line of equal values. It can be seen that three dots, each representing patients, or 14.3% of the patients are not within the surrounding of this line showing an asymmetric expansion with greater movement of the left segment (Fig. 9).

## Discussion

Asymmetric expansions, where one half of the maxilla moves more than the other, have been described as a complication of SARME procedures [18, 24]. Since the bone structures of the midface are generally not symmetrical to each other, the transverse widening, after fixation of the bone-anchored distractor to the palate, is also not symmetrical. A side-dependent difference in the bone stiffness forces an asymmetrical distraction. In most cases of transversal expansion the asymmetry can be compensated by tooth movement. In the worst case, correction of this situation would mean patients have to undergo another surgical intervention [27].

This study investigates expansion behavior regarding symmetry after targeted bone weakening of the maxilla in surgically assisted rapid maxillary expansion.

Pre- and postsurgical CBCTs were superimposed at the anterior cranial base with a 3D measurement software GOM inspect (GOM GmbH, Braunschweig, Germany) in this study by means of a local best fit. This software has proven itself in other studies for superimposition purposes [33, 34].

Superimposition of CBCT models using the anterior cranial base as reference, like in this study, has shown to be accurate. This area is also not affected by the expansion of a SARME and provides accurate results [35].

The treatment method used in this study is based on that of Lines, which was published in 1975 [30]. Lines’ treatment procedure is based on Le Fort I level with an osteotomy starting from the apertura piriformis and extending over the zygomatic columns to the maxillary tuberosity. He emphasizes that a further distal extension up to the pterygomaxillary fissure is not necessary, because this way damage to the pterygoid plexus and the descending palatal artery can be avoided.

Seeberger et al. concluded that a separation of the pterygomaxillary connection can be dispensed during the surgical procedure. However, this leads to an anteriorly accentuated V-shaped expansion due to the posterior resistance zone, so that a separation of this structure was obligatory to allow symmetrical expansion [36]. In two cadaveric studies, Möhlhenrich et al. reported about similar effects of pterygoid disjunction in context of SARME [20, 37].

Our findings confirm this V-shaped expansion pattern as transverse width measured at the buccal cusps of the first upper premolars and molars shows greater expansion in the area of the premolars. The interpremolar width changed by 6.13 ± 4.63mm and the intermolar width by 4.2 ± 4.64mm. Ramieri et al. show an interpremolar change of 6.86 ± 1.5mm and an intermolar width change of 5.39mm ± 2.5mm which seems to be in concordance with our findings and of those published in a literature review by Koudstaal et al. [18, 26]. Negative values of movement as were seen in cluster 2 could be explained by a posterior inward rotation of the lateral segments by this V-shaped expansion pattern. These values could also be affected by positioning of the TPD in anteroposterior position as it has an effect on expansion pattern and on symmetry of expansion [38] and by vertical positioning of the device having an effect on segmental tipping [39].

Another study investigating the clinical effects of TPD demonstrates that the main effect of expansion is rather at level of the alveolar crest than in the maxillary base [40].

Measurements of distances of the root apices to a reference line at level of the midpalatal sutures in this study were less than the transverse width changes at the buccal cusps supporting the fact that expansion is less at the maxillary base compared to areas closer to the occlusal plane.

Regarding angular changes in this study first premolars and first molars showed a more acute angle of their long axis to the occlusal plane at T1 indicating buccal tipping of the teeth. Our findings are not in line with those of Pinto et al. as they even noticed a palatal tipping of the premolars by 8.3° ± 9.6° [39].

A study investigating a tooth-borne SARME found at first premolar level 6.48° ± 2.29° degrees of buccal tipping and at first molar 7.04° ± 4.58° of buccal tipping [41]. By using a bone-borne transpalatal distractor, we could achieve less buccal tipping, but it was still unavoidable. Similar results regarding buccal tipping by bone-borne devices have been described [24].

The amount of skeletal expansion achieved was less than the measurements of the transverse changes in interpremolar and intermolar width. Taking additionally into account that buccal tipping has occurred and that movement of the root apices was also minimal compared to more coronal movement, it has to be concluded that bone-borne devices have dental effects which are not to be neglected.

While investigating skeletal effects, the skeletal landmarks have to be chosen for 3D surface distance measurements. The defined landmarks were similar to another study investigating the expansion behavior of the maxilla, but instead of points, they used rectangular areas of bone for surface comparison [23].

Analyzing the movement of the skeletal segments with the Wilcoxon test globally for all patients revealed no significant difference in expansion of the left and right segment indicating a symmetrical expansion could be achieved. During the surgical procedure, iatrogenic bone bridges are left in the lateral sinus wall which have been varied by an iterative mathematical algorithm in order to achieve a symmetric expansion. This idea contradicts the concept of Kober stating that a more symmetric osteotomy leads to a more symmetrical expansion [42].

Cluster analysis revealed that in 15 patients of our study, there was a significantly higher effect in all three segments to be seen by transverse expansion to be seen than in the other 6 patients (Fig. 9).

A possible reason for this could be the same rationale as described for asymmetric expansion like occlusal factors [24], resistance of tissues [23], or maybe the iatrogenic bridges at osteotomy enhancing higher resistance of hard tissue.

Two-factorial analysis of variance supports the fact that the left and right segments have expanded symmetrically for both clusters even if one cluster had a minor movement of the segments compared to the other.

Analysis of left/right asymmetry within the cluster showed no significant difference indicating symmetric expansion of the left and right segments (*p* = 0.073). The low *p* value suggests looking at the cases individually and might indicate that the amount of distraction might play a role in order to aid symmetry.

It was found that 3 patients showed an asymmetric expansion, which is 14.3% of the sample size. An asymmetric skeletal expansion more than 3mm is considered as clinically relevant, and another study has shown that this occurred in 55% the patients evaluated [23]. Other studies have shown a significant asymmetric expansion in only 13.8% of the patients [42]. It should be taken into account that asymmetric transverse expansion can also be the result of an oblique placement of the transpalatal distractor [26]. An insertion with insertion guides may seem favorable.

From our observations, it can be concluded that there was a retraction of the anterior maxillary region. A similar finding was also made by Nada et al. who additionally reported on possible retroclination of the upper incisors [11].

This is a retrospective study without a predefined sample size or control group and is only of explorative character. The results of retrospective studies are to be interpreted with caution due to the limitations and as risk of bias can not be ruled out. Additional limitations of our study were that asymmetries were only considered in transverse plane and not in any other direction. This study did not take into account relapse movements. A surgical protocol was not predefined, but due to computational workflow with surgical guides, it was similar for all patients. A sample size calculation was not performed but relates to other studies analyzing expansion behavior [23].

Despite these restrictions, this study investigates a novel technique and could be of help for further research regarding development, validation, and provision of evidence for new CAD/CAM techniques in surgical interventions like SARME with targeted bone weakening.

## Conclusion

CAD/CAM/CAE-aided SARME with targeted bone weakening can achieve symmetrical skeletal expansion of the left and right maxillary segments.

Using bone-borne distractors and computational simulation for surgical maxillary expansion does not prohibit dental effects in transverse expansion.
